# Dengue Infection and Miscarriage: A Prospective Case Control Study

**DOI:** 10.1371/journal.pntd.0001637

**Published:** 2012-05-08

**Authors:** Peng Chiong Tan, May Zaw Soe, Khaing Si Lay, Seok Mui Wang, Shamala Devi Sekaran, Siti Zawiah Omar

**Affiliations:** 1 Department of Obstetrics and Gynaecology, Faculty of Medicine, University of Malaya, Kuala Lumpur, Malaysia; 2 Department of Medical Microbiology, Faculty of Medicine, University of Malaya, Kuala Lumpur, Malaysia; Tropical Medicine Institute Pedro Kourí (IPK), Cuba

## Abstract

**Background:**

Dengue is the most prevalent mosquito borne infection worldwide. Vertical transmissions after maternal dengue infection to the fetus and pregnancy losses in relation to dengue illness have been reported. The relationship of dengue to miscarriage is not known.

**Method:**

We aimed to establish the relationship of recent dengue infection and miscarriage. Women who presented with miscarriage (up to 22 weeks gestation) to our hospital were approached to participate in the study. For each case of miscarriage, we recruited 3 controls with viable pregnancies at a similar gestation. A brief questionnaire on recent febrile illness and prior dengue infection was answered. Blood was drawn from participants, processed and the frozen serum was stored. Stored sera were thawed and then tested in batches with dengue specific IgM capture ELISA, dengue non-structural protein 1 (NS1) antigen and dengue specific IgG ELISA tests. Controls remained in the analysis if their pregnancies continued beyond 22 weeks gestation. Tests were run on 116 case and 341 control sera. One case (a misdiagnosed viable early pregnancy) plus 45 controls (39 lost to follow up and six subsequent late miscarriages) were excluded from analysis.

**Findings:**

Dengue specific IgM or dengue NS1 antigen (indicating recent dengue infection) was positive in 6/115 (5·2%) cases and 5/296 (1·7%) controls RR 3·1 (95% CI 1·0–10) P = 0·047. Maternal age, gestational age, parity and ethnicity were dissimilar between cases and controls. After adjustments for these factors, recent dengue infection remained significantly more frequently detected in cases than controls (AOR 4·2 95% CI 1·2–14 P = 0·023).

**Interpretation:**

Recent dengue infections were more frequently detected in women presenting with miscarriage than in controls whose pregnancies were viable. After adjustments for confounders, the positive association remained.

## Author Summary

Dengue is the most prevalent mosquito-borne infection with two billion of the world's population at risk and 100 million infections every year. Dengue is increasingly important due to expansion in the vector's range, increased population density in endemic areas from urbanisation, social and environment change. Miscarriage and stillbirth is associated with dengue when the illness is severe. Dengue can also be transmitted directly from the ill mother through the placenta to the fetus in later pregnancy with variable effect to the fetus. However, dengue infection is asymptomatic to mild only in almost 90% of cases and up to 20% of pregnancies miscarry. Little is known if dengue infection in early pregnancy particularly when it is asymptomatic or mild has an effect on miscarriage. Our study explored the relationship between dengue and miscarriage by looking at recent infection rates amongst women who had miscarried and those whose pregnancies were healthy in an area were dengue is common. Our study finds a positive association between recent dengue infection and miscarriage. This finding may be important in explaining some of the miscarriages in areas where dengue is common. It is also relevant to newly pregnant women from non-dengue travelling to dengue endemic areas.

## Introduction

Each year there is an estimated 100 million dengue infections [1], 500 thousand hospitalisations due to severe dengue illness [2] and 25 thousand dengue deaths [1] worldwide. Dengue's geographical reach is expanding [3] due in part to the complex interplay of climate warming [4] coupled with travel and trade [5] impacting on the observed extension in the range of the responsible mosquito vector [6]. Dengue's intensity in endemic areas has also increased due to the spread of urbanisation [3].

Dengue illness is caused by any one of four serotypes. Infection by one serotype is thought to produce lifelong immunity to that serotype but confers only a few months immunity to the others [7]. Secondary infection by a different serotype increases the risk of severe illness [8]. Typically, dengue infection is asymptomatic or minimally symptomatic in 87% of cases [9]. Hence, obvious dengue illness is the tip of the iceberg in dengue infection.

In Malaysia where dengue is hyperendemic, all four serotype circulate concurrently [10]. In a recent report on a national sample of 1000 Malaysian adults aged 35–74, the dengue seroprevalence rate was 91.6% with a positive age related trend [11].

Severe dengue illness during pregnancy is associated with major adverse outcome of maternal deaths [12], [13], perinatal deaths [12]–[14], preterm births [12], [14] and haemorrhages in labour with much of the data from case reports and small case series [15]. In a large prospective study of recent dengue infection detected at delivery, the pregnancy outcome of 63 parturients who were dengue IgM positive is not different from 2468 IgM negative controls [16]. Vertical transmission to the fetus (particularly later in pregnancy) is established [15]. The vertical transmission rate of maternal dengue infection can vary from at least 1·6% [16] to 6·8% [17] based on prospective studies.

Miscarriages have been reported in association with dengue illness [13], [14], [18], [19] but it is not clear whether the miscarriages were secondary to profound systemic disturbance as a consequence of severe dengue illness or to vertically transmitted dengue infection. Little is known on the effect of dengue infection in early pregnancy with regard to the risk of miscarriage. According to a recent review, pregnancy does not appear to increase incidence or severity of dengue but the literature is very sparse with very few systematic studies of dengue infection on pregnancy available [20].

Dengue specific immunoglobulin M (IgM) can be detected as early as the second day of symptoms and for up to three months [21]. The IgM level typically peaks on the sixth day [22] reaching 100% detection by the eighth day [23]. The dengue IgM test cross-reacts across all four dengue serotypes [24] and is produced even in a secondary infection [25]. The dengue non structural protein 1 (NS1) antigen test is designed to diagnose all serotypes of dengue infection from Day 1 to Day 9 [22]. Dengue IgG antibodies is detected in 100% of cases by Day 15 of primary infection [23] and its presence without other evidence for acute infection is usually taken as distant exposure to any combination of the four dengue serotypes.

We hypothesise that miscarriage is associated with exposure to dengue during early pregnancy due either to maternal dengue illness or vertical transmission. We sought to evaluate the prevalence of recent dengue infection (using dengue IgM and NS1 tests) in women presenting with miscarriage and in similar gestation women with viable pregnancy and to estimate the relative risk of miscarriage in women recently exposed to dengue infection.

## Methods

### Ethics statement

Ethics approval for the study was obtained from the University of Malaya Medical Centre Medical Ethics committee (approval no. 715.25 dated 22^nd^ April 2009). The study was conducted in keeping with the Declaration of Helsinki (amended Seoul, Korea 2008) on human study.

We performed a prospective matched case control study on women who presented to our city-based university hospital with miscarriage and control women attending our hospital for pregnancy related care whose pregnancy was viable. Relevant care providers in our hospital were briefed about the study and requested to contact the research team when they diagnosed a miscarriage at up to 22 weeks gestation so that the patient can be approached and consented for study enrolment. All participants provided their written consent.

### Participants

From 9 June 2009 to 16 March 2011, women who presented acutely to our hospital and diagnosed as a miscarriage were approached. For the purpose of the study, miscarriage is defined as the presence of non-viable product of conception on ultrasound or physical confirmation of expelled products and a positive pregnancy test. In the event of early miscarriage, a positive urine pregnancy test and a history of expelled products of conception is also acceptable if the uterus is empty at presentation. Gestation should not be more than 22 weeks. Gestation is calculated from the reported first day of the last menstrual period and corrected by ultrasound assessment when deemed clinically appropriate to do so. We excluded women with ectopic pregnancy or whose pregnancy was of unknown location. For every case with miscarriage, we recruited three control women with viable pregnancies matched for maternal age (within 3 years) and gestational age (within 3 weeks). The controls were recruited at the earliest opportunity after the index miscarriage case by a researcher (MZS) from amongst women attending for their pregnancy care at our hospital. Miscarriage cases and controls were derived from the same providers and hospital sources.

### Procedures

All participants answered a short questionnaire about whether they had a clinical diagnosis of dengue, made during the current pregnancy or in their lifetime prior to the current pregnancy or a febrile illness, in the last 10 days or at any juncture in their current pregnancy.

Venous blood (5 ml) was collected from each participant and drawn into plain blood bottles. The blood samples were sent directly to the laboratory for immediate processing or kept in a refrigerator at 4°C prior to transfer if the laboratory was closed. The laboratory is based at our university with extensive experience in dengue work.

Samples were discarded if after centrifugation, the supernatant had the appearance of lysed blood. The spun samples were aliquoted and then stored at −70°C. Sera were tested for dengue specific IgM, dengue NS1 antigen and dengue specific IgG. These tests cross react against all four dengue serotypes.

Dengue specific IgM was tested for using an in-house IgM capture-ELISA (enzyme linked immunosorbent assay) test [26]. Samples were considered positive if the ratio of optical density of the positive to negative control was at least two. Panbio Dengue Early ELISA Catalogue No. E-DEN02P (Inverness Medical Innovations Australia Pty Ltd, Queensland, Australia) kits were used as a dengue NS1 antigen capture ELISA test. For dengue IgG detection, we used Panbio Dengue IgG Indirect ELISA catalogue No. E-DEN01G (Inverness Medical Innovations Australia Pty Ltd, Queensland, Australia) kits. The Panbio kits were utilised as per manufacturer's instructions.

We define a recent dengue infection as the detection in sera of dengue specific IgM and/or NS1 antigen.

The miscarriage cases had their chart reviewed up to their discharge from hospital follow up. All included cases must have a confirmed diagnosis of miscarriage at hospital discharge. Cases who did not attend any hospital follow up subsequent to their recruitment into the study or whose latest follow up data were still inconclusive for a diagnosis of miscarriage were contacted by telephone at least three months after their recruitment to confirm the diagnosis of miscarriage with a view to excluding those with a misdiagnosis. Similarly, controls that did not have confirmation of pregnancy viability beyond 22 weeks gestation from chart review because they transferred care elsewhere were contacted by telephone to confirm pregnancy viability beyond 22 weeks gestation.

We had planned to exclude controls that could not be confirmed to have a viable pregnancy beyond 22 weeks as we could not exclude subsequent late miscarriages in them. Our study is designed to evaluate miscarriages up to 22 weeks gestation; it would be inappropriate to have as controls, women who subsequently had miscarriages. We excluded controls who had miscarried after recruitment as we could not exclude a new dengue infection around the time of their miscarriage as no dengue testing was done then.

### Sample size calculation

In a recent study on parturients performed at our hospital, the maternal dengue infection rate as defined by a positive dengue IgM test was 2·5% [16]. Since pregnancy per se has no effect on the acquisition of dengue infection, we expect our control population to have a 2·5% recent dengue infection rate and for the purpose of the sample size calculation we postulate that miscarriage cases will have a 10% recent dengue infection rate. Taking alpha of 0·05, 80% power, 1 to 2 case to control ratio (using PS sample size calculator) [27], 112 cases and 224 controls are required, . As we expect significant attrition in the number of controls due to care transfer to other institutions and hence their drop out from follow up, we increased our planned recruitment ratio to 1 case to 3 controls i.e. at least 112 miscarriage cases and 336 viable pregnant controls.

### Statistical analysis

Data was entered in to SPSS version 15 (SPSS Inc. San Diego CA USA). Comparison (between cases and controls) of the means of continuous variables was by the Student t test, ordinal variables by the Mann Whitney U test and categorical variables by the Chi square test. Fisher exact test is used in place of the Chi Square test for categorical variables if 2 or more cells contain <5 subjects. Multivariable logistic regression analysis, incorporating in the model all characteristics with P<0·05 on bivariate analyses between cases and controls was performed to adjust for these differences in order to establish their independent association with miscarriage. All tests were 2-sided and P<0·05 was taken as a level of significance.

## Results

**Figure 1:**
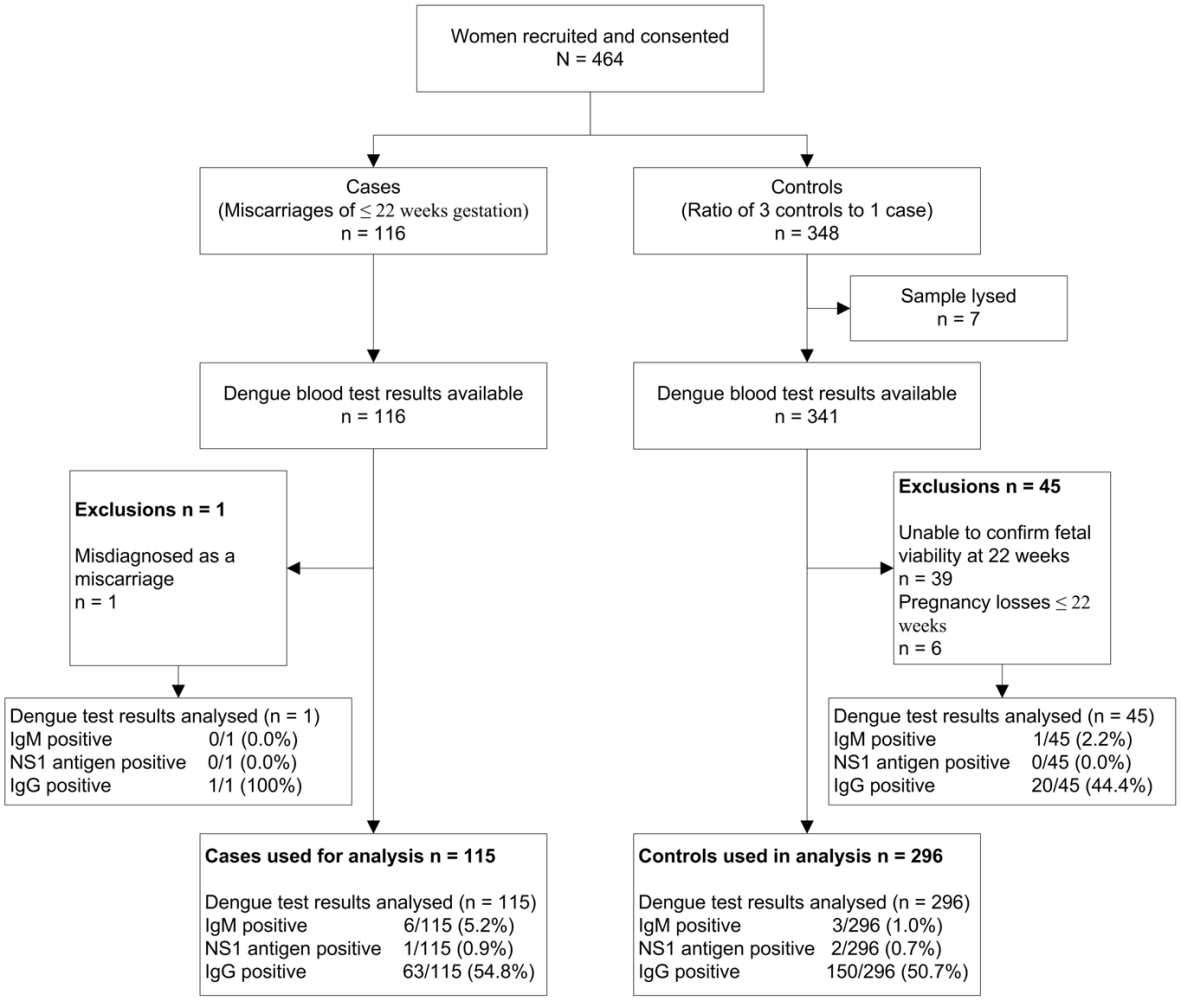
Flow chart of recruits with miscarriages (up to 22 weeks) and controls through the study.

Figure 1 depicted the flow of the recruits within the study and their eventual destination. We recruited and drew blood from 116 cases with a diagnosis of miscarriage and 348 similar gestational aged controls whose pregnancies were viable at recruitment, totalling 464 women for the study cohort. Blood samples from seven controls were found to be lysed after processing and discarded, leaving 457 samples to be tested for dengue IgM, NS1 antigen and IgG. On follow-up, one case of miscarriage was found to be a misdiagnosis and that pregnancy resulted in a term livebirth. Six controls miscarried before 22 weeks gestation and another 39 controls could not be confirmed to have a viable pregnancy beyond 22 weeks gestation despite attempted contact by telephone. These 46 women were excluded from the final analysis.

The characteristics of included cases and controls are shown in Table 1. Prevalence of recent dengue are 5·3% and 1·7%; RR 3·1 (95% 1·0–10) P = 0·047 in cases versus controls. Cases with miscarriages on bivariate analyses were also more likely to be older, of higher gestational age at recruitment and parity and less likely to belong to our major ethnicities of Malay, Chinese or Indian but not significantly more likely to have been ill with a fever during their pregnancy nor more likely to have had distant exposure to dengue (dengue IgG positive status). All the characteristics with P<0·05 on bivariate analysis were included in the model for multivariable logistic regression analysis. After adjustment, recent dengue infection remained an independent risk factor for miscarriage; adjusted odds ratio AOR 4·2 (95% Confidence Interval 1·2–14) P = 0·023.

**Table 1:** Characteristics of a total of 411 cases (confirmed miscarriages up to 22 weeks) and controls (pregnancies that remained viable beyond 22 weeks at follow up).

Characteristics	Cases	Controls	Relative Risk	P value	Adjusted Odds Ratio	Adjusted P value
	n = 115	n = 296	(95% Confidence Interval)		(95% Confidence Interval)∥	
Recent dengue infection (dengue IgM and/or NS1 antigen positive)* †	6 (5·2%)	5 (1·7%)	RR 3·1 (1·0–10)	P = 0·047	AOR 4·2 (1·2–14)	P = 0·023
Dengue IgM antibody positive*	6 (5·2%)	3 (1·0%)	RR 5·2 (1·3–20)	P = 0·009		
Dengue NS1 antigen positive*	1 (0·9%)	2 (0·7%)	RR 1·3 (0·1–14)	P = 0·836		
Dengue IgG antibody positive*	63 (54·8%)	150 (50·7%)	RR 1·1 (0·9-1·3)	P = 0·454		
Maternal age (years)‡ †	31·4±5·7	29·4±4·5		P = 0·001	AOR 1·1 (1·0-1·1)	P = 0·011
Gestation (weeks) at recruitment‡ †	12·4±4·0	11·4±3·5		P = 0·009	AOR 1·1 (1·0-1·2)	P = 0·004
Parity§ †	1 [0–2]	0·5 [0–2]		P = 0·018	AOR 1·1 (0·9-1·3)	P = 0·564
Prior miscarriages (number)§	0 [0–1]	0 [0–1]		P = 0·985		
Ethnicity* †				P = 0·020		
Malay	83(72·2%)	230 (77·7%)			AOR 0·2 (0·1-0·7)	P = 0·005
Indian	14 (12·2%)	41 (13·9%)			AOR 0·3 (0·1-0·7)	P = 0·011
Chinese	5 (4·3%)	15 (5·1%)			AOR 0·2 (0·1-0·9)	P = 0·038
Others	13 (11·3%)	10 (3·4%)				
Diagnosis of dengue infection in life time before current pregnancy*	6 (5·2%)	26 (8·8%)	RR 0·6 (0·3-1·4)	P = 0·226		
Diagnosis of dengue infection during pregnancy*	1 (0·9%)	1 (0·3%)	RR 2·6 (0·2-41)	P = 0·487		
Febrile illness in the 10 days prior to enrolment into study*	8 (7·0%)	12 (4·1%)	RR 1·7 (0·7-4·1)	P = 0·220		
Febrile illness at any time during pregnancy*	8 (7·0%)	23 (7·8%)	RR 0·9 (0·4-1·9)	P = 0·779		

Data displayed as number (%), mean ± standard deviation or median [interquartile range].

*: Analysis using Chi Square test (categorical data).

**†:** Included in logistic regression model.

**‡:** Analysis using Student t test (continuous data).

**§:** Analysis using Mann Whitney test (non-normal data distribution).

**∥:** Multivariable logistic regression analysis incorporating into the model recent dengue infection and adjusting for maternal age, gestational age, parity, and ethnicity, characteristics which are significantly associated with pregnancy miscarriage on bivariate analyses. Maternal age, gestational age and parity were entered as continuous variable and recent dengue and ethnicity as categorical variables.

As a post hoc analysis, if all participants with available dengue test results (n = 457) were analysed, excluding only the seven with lysed samples, the result is not altered: recent dengue infection is still a significant independent risk factor for miscarriage (AOR 3·8 95% CI 1·2–13 P = 0·026).

The 11 participants from both arms of the study group who had recent dengue infection were of similar age, gestational age, parity status, prior miscarriage status, ethnicity and personal awareness of dengue diagnosis in the past but as anticipated, more likely to report a recent febrile illness (as dengue illness is typically febrile) and to be dengue IgG positive (as IgG conversion follows IgM response within a week or two in primary infections) compared to the 400 without evidence of recent dengue – Table 2. These findings support the perception that in our environment with dengue hyperendemicity, dengue is an equal opportunity infection amongst pregnant women.

**Table 2:** Characteristics of women with and without recent dengue infection.

Characteristics	Dengue*	No dengue	Relative risk	P value
	n = 11	n = 400	(95% Confidence Interval)	
Maternal age (years)†	29·4±5·2	30·0±5·0		P = 0·700
Gestation (weeks) at recruitment†	10·8±2·3	11·7±3·7		P = 0·258
Parity‡	0 [0–1]	1 [0–2]		P = 0·631
Prior miscarriages (number)‡	0 [0–1]	0 [0–1]		P = 0·455
Ethnicity§				P = 0·410
Malay	10 (90·9%)	303 (75·8%)		
Indian	0 (0·0%)	55 (13·8%)		
Chinese	1 (9·1%)	19 (4·8%)		
Others	0 (0%)	23 (5·8%)		
Dengue IgG positive	9 (81·8%)	204 (51·0%)	RR 1·6 (1·2-2·2)	P = 0·044
Diagnosis of dengue infection in life time before pregnancy∥	1 (9·1%)	31 (7·8%)	RR 1·2 (0·2-7·8)	P = 0·870
Diagnosis of dengue infection during pregnancy§	1 (9·1%)	1 (0·3%)	RR 36 (2·4–544)	P = 0·053
Febrile illness in the 10 days prior to enrolment into study∥	4 (36·4%)	16 (4·0%)	RR 9·1 (3·6–22·8)	P<0·001
Febrile illness at any time during pregnancy∥	6 (54·5%)	25 (6·3%)	RR 8·7 (4·5–16·9)	P<0·001

Data displayed as mean ± standard deviation, median [interquartile range] or number (%).

*: Defined as women whose sera were dengue IgM positive and/or dengue NS1 particle positive.

**†:** Analysis using Student t test (continuous data).

**‡:** Analysis using Mann Whitney U test.

**§:** Analysis using Fisher exact test (2 or more cells with numbers <5).

**∥:** Analysis using Chi Square test.

Four of the five controls with recent dengue infection reported a febrile illness during pregnancy compared to two such reports amongst the six miscarriage cases. All five controls with recent dengue infection and known outcome proceeded to livebirths at term. In the six miscarriage cases, there was one case of concurrent miscarriage and severe dengue illness. That case required intensive care therapy during her hospitalisation but her miscarriage just preceded the worst of the dengue illness. These findings are not helpful in differentiating whether dengue illness or vertical dengue infection contributed more to the abortion process.

## Discussion

The literature on dengue and miscarriage or spontaneous abortion is sparse. We performed a PubMed (http://www.ncbi.nlm.nih.gov/sites/entrez) search applying terms dengue and miscarriage or dengue and abortion without limitations on October 1, 2011 and retrieved only 11 reports. Of these 11 reports, only four publications involved the study of miscarriages in association with dengue, presenting anecdotal data on only five cases of miscarriage during dengue illness [13], [14], [18], [19]. A recent report on women in refugee camps at the Thai-Burmese border investigating antenatal febrile illness has found a dengue infection rate of 9.5%. One woman (of 20; 5%) who had dengue associated fever during pregnancy subsequently miscarried [28].

Recent dengue infection was found in 11/411 (2·6%) of our study participants, a very similar rate to a recent report from our hospital of 2·5% (63/2531) dengue IgM rate in unselected parturients at time of their delivery [16]; (Chi Square test, P = 0·82). This shows that as a whole, the participants of our current study closely reflect unselected parturients at our hospital with regard to the risk of recent dengue infection and supports the assertion that the selection of our study group is unbiased. The similar dengue IgG positive and reported recent febrile illness rates amongst cases and controls in our current study further bolster the above assertion.

Our dengue IgG positive rate of 213/411 (52%) indicating prior dengue exposure in participants whose mean age was 30 years old is similar to the 63% dengue IgG positive rate from a cross sectional community-based prevalence study on subjects aged 21 to 40 years [29] who were derived from the same geographical catchment area as our participants. This similarity lends support to our study group as a whole being a representative sample of the host population.

It is noteworthy that 6/11 (54·5%) of the women with recent dengue in our sample reported a febrile illness in the course of the pregnancy leading up to their recruitment at a mean gestation of only10·8 weeks whereas in an earlier study at our hospital of 63 dengue IgM positive parturients (delivered at a mean gestation of 39·4 week), only 7/63 (11·1%) reported a febrile illness over the entire course of pregnancy [16]; (Chi Square test P<0·001). Dengue is reported to be asymptomatic or minimally symptomatic in 87% of infections [9], an illness rate similar to that reported by the parturients but far lower than in our current study group. Mild exposures to hyperthermia or fever during the preimplantation period and more severe exposures during embryonic and fetal development often result in prenatal death and abortion [30]. The possible interpretation is that dengue infection in very early pregnancy may give rise to a more symptomatic (or severe) presentation. This more severe effect may contribute to its role in miscarriage whereas dengue infection late in pregnancy is far less likely to be severe and thus did not adversely affect on delivery outcome [16]. However, with only 11 cases of recent dengue found in this study, further corroboration of this hypothesis is required.

Although dengue illness can cause fatigue at two months [31] and other persistent symptoms at two years [32] beyond the acute phase, all five cases of recent dengue infection in our control group resulted in term livebirths despite the fact that four of these five had reported a febrile illness during early pregnancy. The interpretation might be that dengue infection very early on during pregnancy may have a severe impact sufficient to contribute to early miscarriage but does not have a prolonged adverse effect deeper into pregnancy.

There are limitations in our study design. As dengue IgM may persist for up to six months [1] after infection though rarely, our methodology was not capable of identifying only those dengue infections that were within pregnancy – some those identified with recent dengue infection might have been infected prior to pregnancy. Specific IgM might not be detectable in a small proportion of secondary infections [1], [25] and given the dengue IgG detection rate of just over 50% in our participants, probably half of the recent infection we detected were secondary infections. However, these limitations apply to both cases and controls, so should not affect the assessment of relative risk and if anything, would be biased against the finding of a relationship between recent dengue and miscarriage. Our IgM capture ELISA assay for dengue cross reacts with IgM against other flaviviruses notably Japanese encephalitis and Yellow fever viruses but IgM capture ELISA for dengue has demonstrated only a low cross reactivity with Japanese Encephalitis virus [33]. Both yellow fever and viral encephalitis were very rare in Malaysia with a reported rate in 2010 of 0 per 100000 and 0·2 per 100000 population respectively compared to dengue illness rate of 148·7 per 100000 population [34]. With only 11 recent dengue infections identified from a sample of 411 (2·6%), the recent dengue infection rate in our study is somewhat lower than that assumed in our sample size calculation of 2·5% recent dengue infection rate in controls and an assumed 10% in miscarriage cases. As a consequence, although analysis shows a significant association between dengue infection and miscarriage at the 5% level of significance, the relatively small number of infections means that the 95% confidence interval is wide.

In summary, in the context of our setting with dengue hyperendemic, 5·2% of the women with miscarriages displayed evidence of recent dengue infection compared to 1·7% in controls with viable pregnancies (AOR 4·2 95% CI 1·2–14: P = 0·023), a statistically significant and clinically important finding. This interaction between dengue infection in early pregnancy and miscarriage is a highly relevant public health issue due to high number of dengue infections worldwide annually and the typically higher fertility rates in the dengue endemic regions of the world. There is an implication also for travellers from dengue free regions in the early stages of their pregnancies who are journeying to dengue endemic areas. Further study to replicate our data and finding is urgently needed.

## Supporting Information

Checklist S1 **STROBE checklist (case-control).**
(DOC)
